# Supplementary material for: Characterization of CK2, MYC and ERG Expression in Biological Subgroups of Children with Acute Lymphoblastic Leukemia
Source: Int J Mol Sci. 2025 Jan 26;26(3):1076. doi: 10.3390/ijms26031076 (PMC11817342; doi:10.3390/ijms26031076)
Supplement: Supplementary file 1 [file ijms-26-01076-s001.zip › ijms-3335534-supplementary.pdf]

SUPPLEMENTARY MATERIALS

SUPPLEMENTARY METHODS

Validation of *CK2, MYC* and *ERG* expression in thymocytes

Thymocytes were also analyzed as normal internal control about *CK2, MYC* and *ERG* expression respect to Healthy Donors. CK2 expression was absolutely comparable between thymocytes and HDs. Instead, thymocytes FC *MYC* expression was comparable to T-ALL mean FC *MYC* expression. It has been demonstrated the overlapping metabolic rewiring in activated T-cells and *MYC*-transformed lymphocytes due to a series of stimuli (such as IL-7 or IL-2) that support metabolic pathways similar to what happens in tumor cells (with the activation of proto-oncogenes such as c-Myc). Myc expression is attenuated in normal lymphocytes that return to the basal state, but Myc-transformed lymphocytes persistently express Myc, which activates genes involved in glucose and glutamine metabolism. ERG expression was comparable in thymocytes and HDs too (**Supplemetary Figure 8** and **S-Table 3**).

SUPPLEMENTARY TABLES

S-Table 1. Genetic alterations according to *Final Risk Group* in children with T-ALL.

	Non-HR n=15 (%)	HR* n=11 (%)	Total n=26 (%)
PTEN Exon7 deletion or inactivating mutations			
Negative	13 (86.7)	8(72.7)	21 (80.8)
Positive	2 (13.3)	3 (27.3)	5 (19.2)
PICALM::MLLT10			
Negative	14 (93.3)	9 (81.8)	23 (88.5)
Positive	1 (6.7)	2 (18.2)	3 (11.5)
TLX3::HOX11L2			
Negative	8 (53.3)	11 (100)	19 (73.1)
Positive	7 (46.7)	-	7 (26.9)
CDKN2A Δ			
Negative	4 (26.7)	3 (27.3)	7 (26.9)
Positive	10 (66.7)	6 (54.5)	16 (61.5)
Not performed°	1 (6.6)	2 (18.2)	3 (11.6)
IKZF1 Δ			
Negative	10 (66.7)	7 (63.6)	17 (65.4)
Δ1-3	-	2 (18.2)	2 (7.7)
Δ1-8	1 (6.6)	-	1 (3.8)
Not performed°	4 (26.7)	2 (18.2)	6 (23.1)
high-CRLF2			
Negative	9 (60)	10 (90.9)	19 (73.1)
Positive	5 (33.3)	1 (9.1)	6 (23.1)
Not performed°	1 (6.7)	-	1 (3.8)

Table Legend: *according to Final risk group; °Not performed because of lack of diagnostic samples.

S-Table 2. Genetic alterations according to *Final risk group* in 35 childhood B-ALL patients

	Non-HR n=16 (%)	HR* n=19 (%)	Total n=35 (%)
BCR::ABL1			
Negative	16 (100)	11 (58)	27 (77)
Positive	-	8 (42)	8 (23)
ETV6::RUNX1			
Negative	-	19 (100)	19 (100)
Positive	16 (100)	-	16 (100)
MLL/KMT2A rearranged			
Negative	16 (100)	15 (79)	31 (89)
Positive	-	4 (21)	4 (11)
"Others" (without known translocations)		7 (36.8)	7 (20)
CDKN2A-Δ			
Negative		5 (71)	5 (71)
Positive		2 (29)	2 (29)
IKZF1-Δ			
Negative		5 (71)	5 (71)
Positive		2 (29)	2 (29)
high-CRLF2			
Negative		5 (71)	5 (71)
Positive		2 (29)	2 (29)

Table Legend: *according to Final risk group; ° Not performed: not enough samples availability.

S-Table 3.

	CK2 expression		*MYC* expression		*ERG* expression	
	Range	*Mean/Median*	*Range*	*Mean/Median*	*Range*	*Mean/Median*
Thymocytes	-	1.100 / 1.100	-	5.800 / 5.800	-	5.670 / 5.670
Healthy Donors	0.220-3.180	1.237 /0.955	0.298-2.966	1.280 / 0.970	0.222-7.438	2.605 / 2.124
T-ALL	0.546-11.471	4.286 / 3.911	0.475-33.855	6.802 / 4.263	3.372-105.164	38.947 / 30.077
B-ALL	0.499-5.489	3.011 / 2.777	0.326-15.807	2.881 / 1.925	2.981-477.713	191.977 / 180.995

S-Table 4

Gene target	*Primer* sequence (5'-3')	Expected amplicons size
CK2α F	TCATGAGCACAGAAAGCTACGA	158 bp
CK2α R	AATGGCTCCTTCCGAAAGATC	
c-MYC F	CACCAGCAGCAGCGACTCTGA	63 bp
c-MYC R	GATCCAGACTCTGACCTTTTGC	
ERG F	CTCCTCCAGCGACTATGGA	57 bp
ERG R	GCGGCTGAGCTTATCGTAGT	
PTEN Exon7 F	GCTTGAGATCAAGATTGCAGATACAG	446 bp
PTEN Exon7 R	GTCTCACCAATGCCAGAGTAAGCA	
TP53 F	GCGCCATGGCCATCTACA	511 bp
TP53 R	GTTGGGCAGTGCTCGCTTAGT	
RPS6 F	TTCAGCTGCTTCAAGATGAA	750 bp
RPS6 R	CTGACTGGATTCAGACTTAGAAGT	
TLX3/HOX11L2 F	GCGCATCGGCCACCCCTACCAGA	244 bp
TLX3/HOX11L2 R	CCGCTCCGCCTCCCGCTCCTC	
PICALM S1770	GCAATCTTGGCATCGGAAAT	440 bp / 380 bp
MLLT10 AS559	CGATCATGCGGAACAGACTG	
MLLT10 AS1002	GCGCTTCAATGATCCAGATATAGAG	

Expression Data

- *CK2* expression in T-ALL cases vs CEM: Median Fold Change (MFC) 4.129 vs 5.040;
- T-ALL patients [range fold change (RFC): 0.546-11.471] *vs* HDs (RFC: 0.350-2.180);
- non-HR-T-ALL *vs* HDs: MFC 2.281 *vs* 0.955, (p=0.0105);
- HR patients *vs* HDs: MFC 5.893 *vs* 0.955, (p=0.0003);
- 4 out of five PTEN *Exon7* mutated vs wild-type: MFC-*CK2* 4.469 *vs* 2.626 (p=0.260);
- *MYC* expression in CEM-cell line vs T-ALLs: MFC 7.463 vs 6.802;
- In cases with PTEN mutated vs wild-type: MFC-*MYC* 16.799 and RFC-*MYC* 7.822-33.851;
- In cases with overexpression of CRLF2 vs normal expression: MFC-*MYC* 16.404 vs 3.921;

SUPPLEMENTARY FIGURES

S-Figure 1

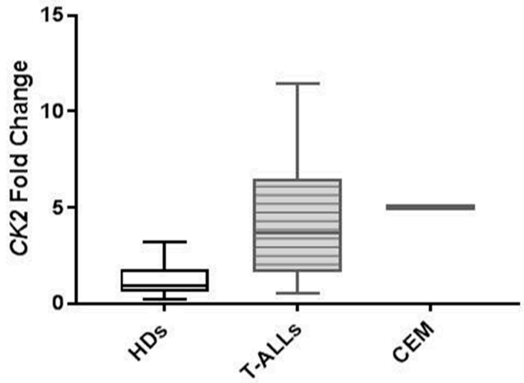

Supplementary Figure 1. CK2 mRNA expression in CEM-cell line was comparable with T-ALLs (mean fold change 5.040 Vs 4.129, respectively) Vs HDs (mean fold change 1.244)

S-Figure 2

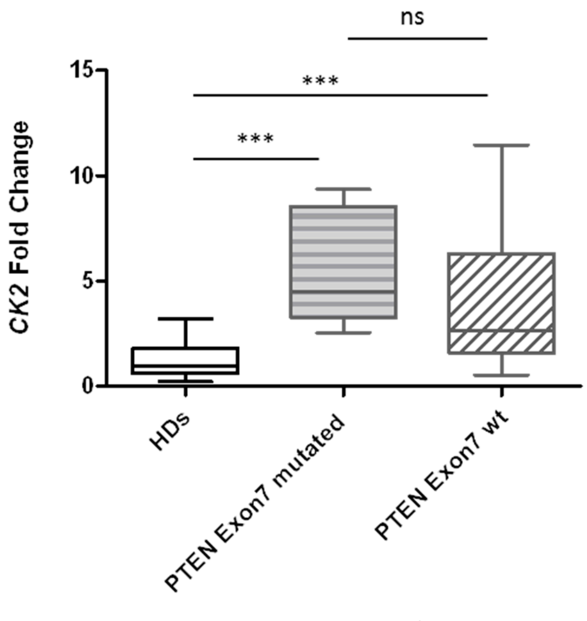

Supplementary Figure 2. Four out of 5 PTEN *Exon7* mutated T-ALL patients showed high CK2 mRNA level (mean=5.621) though it was not significantly different between PTEN-wild type (wt) and mutated samples. CK2 expression was statistically different comparing both PTEN Exon7 wild type and mutated patients to healthy donors

S-Figure 3

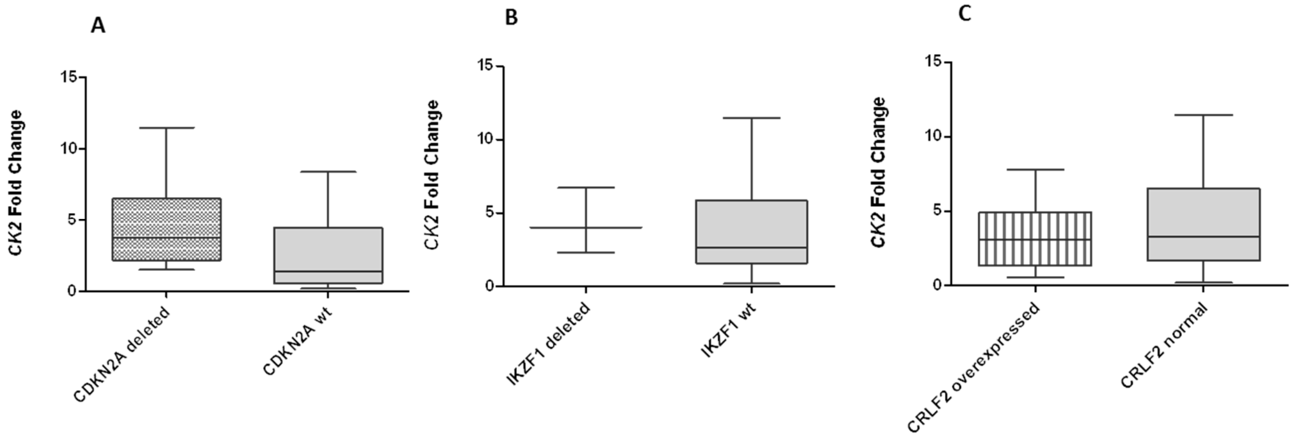

Supplementary Figure 3. The sixteen *CDKN2A* deleted patients (**A**) and the three *IKZF*1 deleted patients (**B**) showed high-*CK2* mRNA levels, although data is not statistically significant (Mean *CK2-CDKN2A* deleted 4.318 Vs Mean *CK2-CDKN2A* wild type 2.498; Mean *CK2-IKZF*1 deleted 4.342 Vs Mean *CK2-IKZF*1 wild type 3.930). No difference about *CK2* expression among *CRLF*2 overexpressed and normal expressed (Mean 3.334 Vs 4.170) (**C**).

S-Figure 4

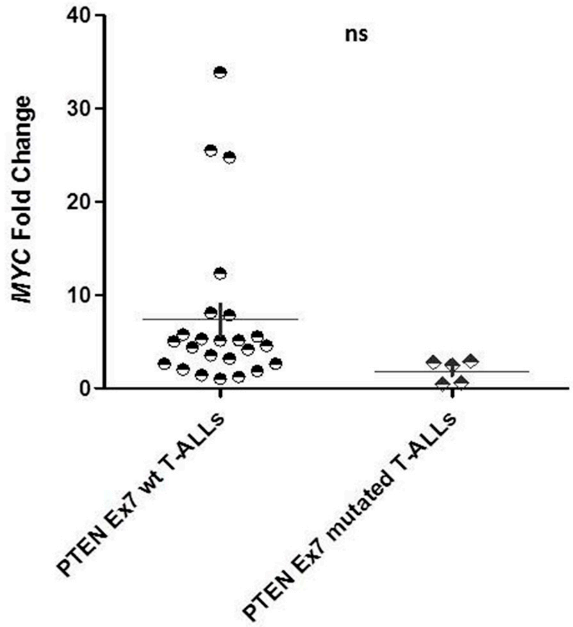

Supplementary Figure 4. PTEN *Exon7* mutated patients showed a low expression of *MYC* compared to PTEN wild-type: MFC 1.838 (range 0.475-2.871) vs 7.984.

S-Figure 5

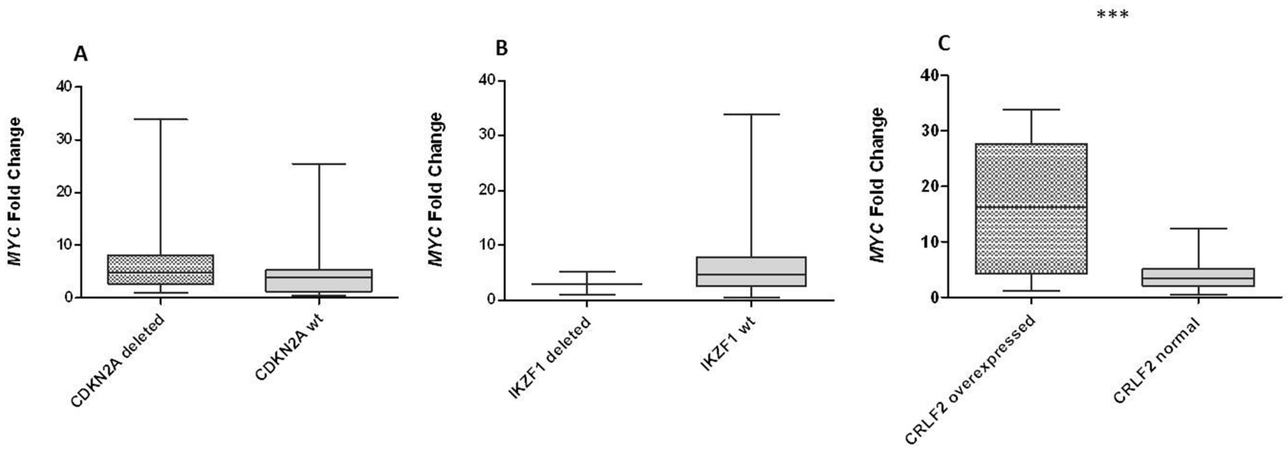

Supplementary Figure 5. *MYC* expression difference between ~~both~~ *CDKN2A* and *IKZF*1 deleted and wild type patients: no statistically significant difference (**A** and **B**). By contrast, comparing *CRLF*2 expression to *MYC* expression, it is showed that *CRLF*2 overexpressed patients presented higher *MYC* expression than *CRLF*2 normal expressed (mean FC MYC expression 16.404 Vs 3.921, respectively) (**C**)

S-Figure 6

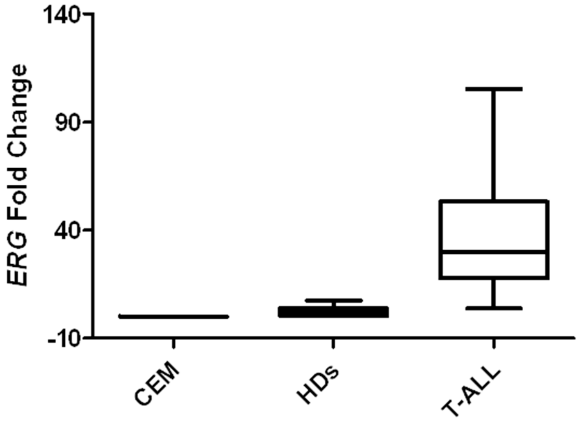

Supplementary Figure 6. *ERG* expression in CEM-cell line was very low, compared to HDs and T-ALL cases, respectively [*ERG* MFC CEM 0.100 *vs* *ERG* MFC HDs 2.605 *vs* *ERG* MFC T-ALLs 38.497]

S-Figure 7

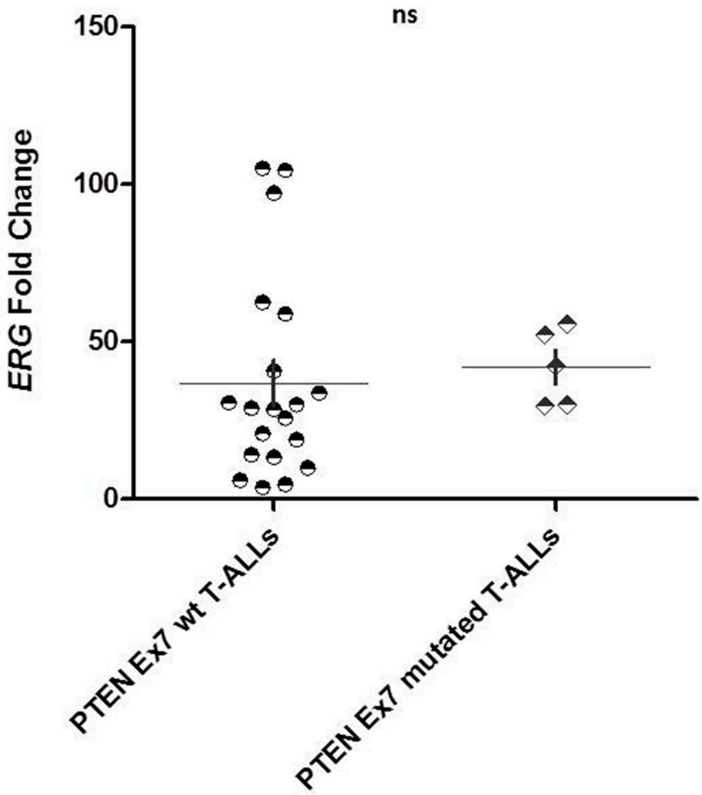

Supplementary Figure 7. The five PTEN Exon7 mutated T-ALLs showed slightly higher levels of ERG mRNA (*ERG* MFC PTEN Exon7 wild type 38.052 Vs *ERG* MFC PTEN Exon7 mutated 41.973)

S-Figure 8

A **B** **C**

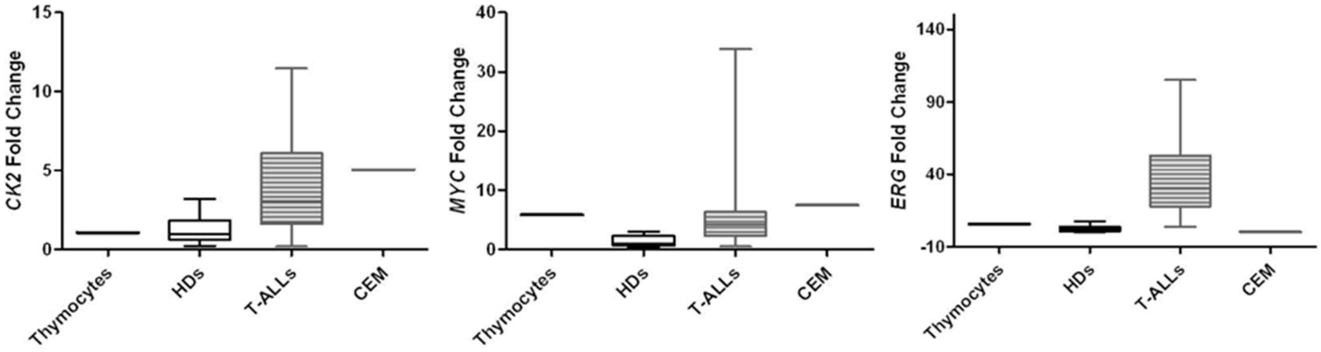

Supplementary Figure 8. *CK2* (A), *MYC* (B) and *ERG* (C) expression in Thymocytes. Healthy donors, T-ALL cells and CEM.
